# Patients with advanced cancer in Uganda: Gender, social norms, and family relationship icebergs in the face of terminal illness

**DOI:** 10.1017/S1478951524002189

**Published:** 2025-01-21

**Authors:** Julia D Kulikowski, Eve Namisango, William E. Rosa

**Affiliations:** 1 Department of Psychiatry and Behavioral Sciences, Memorial Sloan Kettering Cancer Center, New York, NY, USA; 2 African Palliative Care Association, Kampala, Uganda

**Keywords:** Cancer, gender norms, gender-based violence, palliative care, Uganda

## Abstract

**Objectives:**

Cancer is associated with physical, social, spiritual, and psychological changes in patients and their caregivers. However, in sub-Saharan Africa, there is lack of evidence on the impact of gender, social norms, and relationship dynamics in the face of terminal illness. The aim of this paper is to explore how gender identity, social norms, and power relations are impacted when a person is living in Uganda with advanced cancer.

**Methods:**

Focus groups with adult men and women living with advanced cancer in Uganda were conducted. Interviews explored the social effects of cancer and common challenges, including how both disease and treatment affect the patient and marital relationships within their families. Participants’ recommendations were sought to improve the social well-being of patients and their families. Data were analyzed using inductive thematic analysis.

**Results:**

Men and women experienced negative changes in their roles and identities, often feeling unable to fulfill their marital duties in terms of intimacy, their social roles and responsibilities based on societal expectations. Men expressed loss of a “masculine” identity when unable to provide economically for the household. This led to tension in the familial power dynamics, contributing to relationship breakdown and gender-based violence (GBV) against spouses. Women noted challenges with parenting, relationship breakdowns, and increased GBV.

**Significance of results:**

Gender impacts the patient and the family dynamic throughout the life course, including during advanced cancer. Patients and caregivers experience a change in their roles and identities while coping with existential distress and end-of-life tasks. Given these results, gender considerations and dynamics should be incorporated into overall palliative care provision. In addition, there is a need to integrate GBV screening and support in cancer services to address social health and safety needs in the context of serious illness.

## Introduction

Cancer is one of the most common life-limiting illnesses around the globe and 1 of the 3 leading causes of premature death in Africa (Bray and Parkin 2022) with significantly increased prevalence projected as the African continent sees its fastest population growth between 2015 and 2030. The 2022 Global Cancer Observatory from the World Health Organization (Ferlay et al. 2024) reported a total of 763,843 cancer-related deaths in Africa with the highest mortality associated with prostate cancer in men and breast cancer in women (Sung et al. 2021). The risk of death is higher in women with greatest mortality associated with breast, cervical, and uterine cancers (Sung et al. 2021).

Advanced disease states bring significant psychosocial challenges for patients, marital dyad and families, often characterized by existential distress and changes in roles and identity. In many regions of the world, but particularly in contexts like Africa where palliative care access is limited, caregivers play a significant role in caring for family with advanced cancer (Global Atlas of Palliative Care (2nd edn) 2020). Caring for a loved one with advanced cancer has been shown to create a unique set of complex dynamics with changes in gender roles and social norms with women experiencing a higher burden of psychosocial strain and impact on their families and finances (Coles et al. 2024; Ferrant et al. 2014; Ginsburg et al. 2023).

In Africa, there is a deficit of literature exploring the impact of gender, social norms, and relationship dynamics in the face of terminal illness. This study sought to fill this evidentiary gap. Specifically, the study explored how gender, social norms, and power relations in a marital dyad and family unit are impacted when an individual is living with advanced cancer in Uganda (see Table 1 for background on the Ugandan context). Following a description of study findings, this paper discusses aspects of end-of-life care including experiences of the patient, gender differences, and, more broadly, the limited access to palliative care and the impact on regional and global policy.

**Table 1.** Uganda: relevant background and data

Uganda is a landlocked country in East Africa bordered by Kenya, Tanzania, Rwanda, the Democratic Republic of the Congo, and South Sudan. The population of Uganda is 44.4 million and has a life expectancy of 68 years (World Health Organization (WHO) 2024a; 2024b). Uganda has significant burden of noncommunicable disease (NCD) which accounted for 36% of deaths in 2019 (World Health Organization (WHO) 2023). The greatest risk of death from NCDs includes cardiovascular disease and cancer (World Health Organization (WHO) 2023). Uganda is seeing a growth in NCDs in an already overburdened health-care system (Kalyesubula et al. 2019) with limited budget allocation for screening and primary preventative measures (Uganda Government 2018). Overall, the most common causes of death include neonatal conditions, HIV/AIDS, and lower respiratory infections. The health-care system in Uganda is made of a portion of private and public funded facilities. The majority of palliative care services are accessed through public hospitals (Kagarmanova et al. 2022). Over the past 3 decades, the availability of palliative care services in Uganda has grown since a model of care was established in 1993 by Dr. Anne Merriman and Fazal Mbaraka (Merriman and Harding 2010); however, it is estimated that only 10% of the need is met (Kagarmanova et al. 2022).

## Methods

This qualitative study was conducted from May to June 2021 at Hospice Africa, Uganda. Participants were male or female patients with advanced cancer, aged 18 years or older, maintained sufficient cognitive ability to give informed consent and engage in the discussion, and were willing to participate in palliative care related research projects. Exclusion criteria excluded those who had insufficient English or Luganda to participate in the focus group discussion or were too ill to participate.

### Recruitment

The study participants were identified through Hospice Africa in Uganda. This study was reviewed and approved by the Hospice Africa Uganda Research and Ethics Committee (Ref HAU-2021-4). Potential participants were briefed about the planned workshop and those interested in participating were referred to the principal investigator (E.N.) for further details. The principal investigator followed up with the potential participants to provide further details about the study and subsequently obtained informed consent. Once informed, consent was obtained, the date and venue of the workshop was communicated.

### Data collection

We conducted 2 focus group discussions in May 2021, one group comprised of men and the group of women, with 10 participants each. The participants were asked about how cancer affected them socially and explored common challenges. The discussions were guided by a set of questions, which covered broad topics (Table 2). Other questions included how the disease and treatment affected the patient and their family/caregiver and the quality of their relationships. The group facilitator also asked the groups for their recommendations of ways to improve the social well-being of patients with advanced disease and their families.

**Table 2.** Focus group discussion questions

What are the major psychosocial concerns in living with cancer? How did your social /psychosocial life change following the cancer diagnosis? What gender and social norms shape the way you experience life as a cancer patient and your families? What changes have been difficult to cope with since your cancer diagnosis? What could help improve your psychosocial/social well-being to live in the new normal of the life with a diagnosis? Please share some stories based on your experiences and what happened during your illness. How did you cope with challenges and what helped most?

The duration of each focus group was approximately 75 minutes and facilitated by a palliative care provider of the same gender as the participant group. Each group included a note taker who directly made observational notes throughout the group discussion. Prior to the focus group, each participant completed a basic demographic form (age, gender, and diagnosis). All patients were receiving palliative care under Hospice Africa Uganda and had advanced disease.

### Data management and analysis

The study adhered to the Consolidated Criteria for Reporting Qualitative Research (COEQ) guidelines for a comprehensive framework for reporting focus group data (Tong et al. 2007). The recordings were stored on a password protected computer to be used for education and knowledge translation purposes only. The audio-recordings were transcribed verbatim. The transcripts were imported into NVivo 12 and analyzed using thematic content analysis (Braun and Clarke 2006, 2022; Krippendorff 1980), performed by 2 study team members trained in qualitative data analysis (E.N. and N.B.). E.N. read all transcripts and generated inductive descriptive and interpretive codes, while N.B. followed the same process for a subset of transcripts. They then met to reach consensus on code names, descriptions, and potential applications, resulting in a codebook. Using the codebook, they independently coded each transcript using NVivo 12 software (Lumivero, Denver, CO), highlighting significant statements. The coding team met regularly to reach consensus regarding code application, emergent codes, and resolution of discrepancies in coding. In the final phase, the team reviewed code reports to identify key themes observed across transcripts. They then produced a thematic matrix that was reviewed by 2 patient participants, known as member checking. The patient participants provided input on the thematic interpretation of the data, as well as the selection of illustrative quotations.

## Results

Table 3 summarizes the sociodemographic characteristics of the men and women involved in the discussion groups. All individuals were between the age of 18 and 78 years. Cancers in the male group included prostate at the highest frequency followed by Kaposi sarcoma, penile, and esophageal cancer. In the female group, cervical cancer was highest in frequency followed by breast and liver cancer.

**Table 3.** Sociodemographic characteristics of group participants

Characteristic	Variable	Frequency
**Gender**	Male	10
	Female	10
**Age-group (years)**	18−24	2
	25−35	8
	36−45	11
	>45	9
**Marital status**	Single	4
	Married	6
	Divorced or separated	5
	Widowed	3
	Cohabitating	2
**Type of cancer**	**Male**	
	Prostate	4
	Kaposi Sarcoma	2
	Penile	1
	Esophageal	1
	Liver	2
	**Female**	
	Cervical	5
	Breast	4
	Liver	1

Themes that emerged included loss of dignity, alterations in family dynamics, changes in gender roles, financial strain, and changes in intimacy. Tables 4 and 5 show direct quotations from the both discussion groups with the most significant and common findings. Both men and women noted a negative change in their role in the family and marital dyad feeling unable to fulfill intimacy needs of their partner, their social roles and responsibilities based on societal expectations. Men expressed loss of a “masculine” identity following the loss of their ability to provide economically for the household contributing to financial strain. Because of this change, men often felt the power dynamics change with their partner which led to the breakdown of a relationship and gender-based violence (GBV) against spouses. Women reflected on challenges with parenting, relationship breakdowns, and increased GBV. Common experiences included loss of dignity, alterations in family dynamics, and changes in roles as well as intimacy and financial challenges.

**Table 4.** Quotation table from interviews with men

Quotation number	Relevant finding	Quotation
1	Loss of dignity Alternations in family dynamics Relationship breakdown Changes in roles Financial challenges	“The rude health workers contribute to the damaging of our family relations. The caregivers get angry, fed-up and at times quit. My wife spent 9 months with me at the Uganda Cancer Institute and once I was discharged, she packed her belongings and left our marital home and never came back. My wife brought the children to men when we divorced, I was very ill and helpless. This may cause someone to give up on life. I do not blame her but I blame the health workers who were so rude. I lost of money because I had just been introduced to her the family. Even right now, she returns to visit them but still wants to give orders and this causes tension in the family.”
2	Loss of dignity	“The person loses dignity because of the way they are handled by staff. In-fact If a person dies he or she should be handled well as a human being. They carry corpses like garbage and dump them in refuse bags. This is very painful, it hurts the caregivers and also distresses the patients”
3	Alternations in family dynamics Relationship breakdown Intimacy challenges	“I separated with my wife, because I developed chronic fatigue and could not satisfy sexually and also I could no longer work effectively. I just prefer to sit for most of the time our situation is difficult
4	Alternations in family dynamics Relationship breakdown Changes in roles Financial challenges	Also, because I was struggling financially, and I was losing assets to take care of the medical needs. So, things worsened she realized I was selling off everything in the home. I can tell you most marriages have broken down. Even performing their duties as women, they stop because they are fed up and also you are useless as a man. She cannot even wash your shirt. She will go visiting and return late and then you cannot say anything because you have no money. Ideally a man should remain the head of the family as a man, so you ponder and wonder, because you cannot do it anymore. Then also remember when there is disagreement in the home, the children will side with their mother.”
5	Alternations in family dynamics Relationship breakdown Changes in roles	“Personally, my wife left because of this problem and since then I have never married again. Even my children started resenting me, because I was not getting well and they were no longer attending school. If you want peace in the home, you look at the wife as a sister then she will take care of you, but if you insist on being a husband, there will be no peace in the home but if you use the trick I have given you, she can even help you with transport and can buy some fish and make you a good meal. You may also call your old friends and they rescue you with some transport.”
6	Financial challenges	“The woman can even ask if you married other women at the Uganda Cancer Institute or you got a job there. We are very poor, very poor, we can no longer afford the medication if it requires purchase. I think poverty and the poor patient handling just demean humanity.”
7	Loss of dignity Financial challenges	“Even if the woman is working, she cannot help with rent costs, so this disease presses you as an individual, your family and relationships. Covid 19 has further worsened the situation, you cannot ask for help from anyone because people have lost jobs. You go without meals and yet you have medication to take. I can tell you the situation is tense, so patients die of psychosocial distress. Even if there are community level projects, we are excluded because they think we are dying soon, but is this fair? No one is going to carry the project on his or her head.”
8	Loss of dignity Alternations in family dynamics Relationship breakdown Changes in roles	“Men are the household heads, but when they have no money, and can no longer work, there is disability. You have children who must go to school and also feed. Then you are stuck, and your esteem is no more. You can run away from the home if you are not s strong person.”

**Table 5.** Quotation table from interviews with women

Quotation number	Relevant finding	Quotation
1	Alternations in family dynamics Parenting challenges, relationship breakdown Changes in roles	“The moment my husband knew about my diagnosis he me chased out of the house, he also refused to give support, when I asked for financial support to buy medication he said -I have no money, cancer is incurable. So he I had call my elder sister who picked me from home and took me to her home. Now its her and my children looking after me. I was also visited by community members and they encourage me. I lost a lot of weight and it was not easy to convince myself that I would be okay.”
2	Alternations in family dynamics Parenting challenges, relationship breakdown Changes in roles Intimacy challenges	“I used to stay home alone, helpless and the children were home, and so young. My husband used to work from a distance, so we had a family meeting and decided to separate, he took me back to the village and Jinja and he only agreed to give me support. I live with one of my children and I then one of my aunties is taking custody of the others. I am not sure if we have separated may be if my cancer cures there might be chance, but we have not had any sexual intercourse since, so I think we separated. But also if it is ca cervix one is happy to separate with the man.”
3	Alternations in family dynamics Parenting challenges Changes in roles Financial challenges	“For me I am doing petty things, I am not a salary earner, so the earning is difficult. Now when you are ill the man goes away, I have my children to look after and also I have to meet the medical bills. I donot have the money. I think to help women, we should also address the issue of money. If you have no money, then the sickness is a big threat. If you have some money, you can help the family. So you become puzzled and the brain is exhausted of thinking. That is how you get other diseases.”
4	Alternations in family dynamics Parenting challenges Changes in roles Financial challenges	“My husband was incarcerated and at the time I was diagnosed, I was on my own and yet I had a four year old child. I was so worried and felt I could not take this. I had no money and requested to only receive supportive care I lost the will to live because I was on own.”

Participants provided recommendations on how to improve the social well-being of patients and their families. The most frequent recommendations included obtaining additional support for children, integrating social support services throughout cancer care, and ensuring there were additional opportunities for socioeconomic strengthening, such as income generation. Support through ongoing counseling was also recognized as key to well-being. Specifically, participants expressed a need for both couples and crisis counseling,

## Discussion

This study aimed to explore how gender, social norms, and power relations in a marital dyad and family unit are impacted when an individual is living with advanced cancer in Uganda. Our study demonstrates that advanced cancer negatively impacts the patient and carries significant adverse consequences on the dyad and family unit. More evidence is required to increase awareness to help build and inform regional and global policy in order to facilitate growth and access to support.

Our study found several common psychosocial challenges that men and women face when living with advanced cancer. These themes included loss of dignity, changes in gender roles, alterations in family dynamics in terms of parenting and relationship breakdowns, intimacy issues, and financial strain. Women most commonly discussed parenting issues, relationship breakdowns, financial strain and GBV, while men discussed loss of a “masculine” identity, relationship breakdowns, and financial strain.

In our focus groups, both men and women identified that they experienced a negative change in their identity and sense of self which has the potential to develop into feelings of loss of dignity. Literature has established that individuals with advanced cancer experience physical, psychological, and social forms of suffering which translates into elevated rates of adjustment disorders, depressive disorders, anxiety disorders, demoralization, and other trauma and stressor-related disorders compared to the general population (Breitbart et al. 2021). Though our study did not focus on the rates of psychiatric disorders, loss of identity, and negative alterations in how individuals view themselves are important factors that may contribute to elevated rates of these disorders and further suffering in individuals with advanced cancer in Uganda.

In our discussion groups, financial strain was an important issue to both men and women with advanced cancer. Financial strain appeared to stem from changes in traditional gender roles, with disease limiting one’s ability to work and additionally as a result of health-care services. In dyads with tradition roles, men who were unable to work, due to disease, experienced distress related relationship breakdowns, and separation as well as negative views of themselves contributing to loss of dignity.

An additional theme that stems from changes in gender roles is alterations in family dynamics, specifically relationship breakdown and intimacy issues. Drawn from our data, these are significant factors that can contribute to GBV. GBV is a significant issue in Uganda with 95% of women found to have a lifetime history of physical or sexual violence as reported from the National Survey on violence in Uganda (Uganda Bureau of Statistics 2021). Violence against women is a critical public health problem and violates the human rights of women (United Nations 1993). There are many risk factors associated with GBV and includes factors at the individual, family, community, and society level (see Table 6). Cancer alone is a risk factor for IPV in women (Coker et al. 2017) and Roberts and colleagues (2016) found that cervical and vulvar cancers in particular are associated with an increased likelihood of lifetime IPV. Violence on to women leads to negative health outcomes and a significant burden on social and economic costs on society (Ginsburg et al. 2023; Knaul et al. 2018). Women experience higher rates of depression, trauma and stressor-related disorders, anxiety disorders, and suicide attempts in addition to smoking, substance use, and risky sexual behaviors with greater mortality and morbidity in children of GBV (World Health Organization (WHO) 2024a; 2024b, Ng et al. 2023). Overall, there are limited studies on IPV in women with cancer, the most recent from Johnson and Pieters (2016) warranting further advocacy and awareness. Unfortunately, women are underrepresented in cancer care, research and policymaking, thus allocation of funding, and research is not prioritized (Ginsburg et al. 2023). Our study demonstrated that in those with advanced cancer, women commonly experienced relationship breakdowns, changes in roles, and intimacy issues which draw comparison to known literature.

**Table 6.** Risk factors associated with gender-based violence (GBV) (World Health Organization (WHO) 2024a; 2024b)

Risk factors for both intimate partner and sexual violence include lower levels of education (perpetration of sexual violence and experience of sexual violence); a history of exposure to child maltreatment (perpetration and experience); witnessing family violence (perpetration and experience); antisocial personality disorder (perpetration); harmful use of alcohol (perpetration and experience); harmful masculine behaviors, including having multiple partners or attitudes that condone violence (perpetration); community norms that privilege or ascribe higher status to men and lower status to women; low levels of women’s access to paid employment; and low level of gender equality (discriminatory laws, etc.). Factors specifically associated with intimate partner violence include past history of exposure to violence; marital discord and dissatisfaction; difficulties in communicating between partners; and male controlling behaviors toward their partners. Factors specifically associated with sexual violence perpetration include beliefs in family honor and sexual purity; ideologies of male sexual entitlement; and weak legal sanctions for sexual violence.

Finally, education and building capacity of palliative care is an integral factor in providing palliative services. One discussant described negative interactions with health-care providers and many spoke to the burden of symptoms causing significant functional changes. The underlying cause of these experiences are likely multifactorial though one should consider the impact of limited resources, insufficient staffing, and minimal palliative training, highlighting the need for more education and increased support for health-care workers and systems to improve the treatment for patients with advanced disease. Participants’ suggestions for improved cancer care included additional forms of support, individual and couples counseling, financial guidance, and care and support for children. Our participants described the consequences of financial strain on an individual and family. These consequences further support the need for greater outreach and access to palliative services to help lessen the many profound psychosocial issues that arise for individuals with advanced cancer. Within these efforts, it is imperative that gender-based supports and GBV screening become integrated into these services to protect women’s rights and provide safety.

This study has several limitations. Our study had a small sample size of a total of 20 participants with 2 focus groups in 1 palliative care center in Uganda. Because of this small sample in 1 geographical location and cultural context, this study cannot be generalized to greater Uganda or Africa. Data were collected from 2021 and may benefit from more recent participant reflection. We would suggest further research to study a greater number of individuals over a longer period of time to gain a better understanding of psychosocial issues that arise in advanced cancer care. We would also suggest to future studies that the investigators approach research in a manner that is culturally sensitive to that location. Local culture is heterogeneous and care at the end of life should be distributed in line with traditional values and practices of the local culture (Ntizimira et al. 2022). For growth or implementation of palliative care services, policy and change must be carried out in a culturally sensitive manner in line with the people in the region for it to be effective and a helpful means of support. Building on the dearth of literature focused on the psychosocial implications of individuals living with advanced cancer can help build awareness of limited access to care and the importance of treatment and support to those with life limited illnesses. Additionally, this can bring about change to region and global policy to help improve gender and access disparities.

In summary, advanced cancer impacts the patient, dyad and family at multiple levels and elicits physical, psychological, and social forms of suffering. This study gives insight into some of the changes that men and women with advanced cancer face in Uganda. Building an awareness and understanding of the impact of advanced illness states on gender roles and identities in the ill and dyad will help to enhance how end-of-life care is implemented in a means that is culturally safe and meaningful. We broadened the scope of this paper to understand the impact on a socio-economic and global policy level to help inform and with an aim to improve cancer care, gender equity, and the profound deficit of palliative care services around the globe.

## Data Availability

The participants of this study did not give written consent for their data to be shared publicly, so due to the sensitive nature of the research, supporting data is not available.
